# Evaluation of a Novel Bioresorbable Device for Guided Bone Regeneration: An In Vitro CBCT Study on Pig Jaws

**DOI:** 10.1155/ijod/2617319

**Published:** 2025-10-31

**Authors:** Stefan P. Bienz, Philippe C. Deggeller, Riccardo D. Kraus, Marina Green Buzhor, Daniel S. Thoma, Ronald E. Jung

**Affiliations:** ^1^Clinic of Reconstructive Dentistry, University of Zurich, Zurich, Switzerland; ^2^Department of Chemistry and Applied Biosciences, Swiss Federal Institute of Technology, Zurich, Switzerland

**Keywords:** biocompatible materials, biopolymer, bone substitutes, cone-beam computed tomography (all MeSH terms); guided bone regeneration, dental implants

## Abstract

**Background:**

To compare the volume stability of guided bone regeneration (GBR) on peri-implant bone dehiscence defects during flap closure using a novel bioresorbable device with a deproteinized bovine bone mineral (DBBM) or an L-shaped DBBM with 10% collagen (DBBM-C).

**Methods:**

A bioresorbable device was designed and printed using a medical-grade polyglycolide-colactide copolymer (PGLA). Twenty peri-implant box-shaped bone defects were created in 10 pig mandibles. GBR procedures were performed with: device (device + DBBM + collagen membrane) or L-shape (L-shaped DBBM-C + collagen membrane + fixation pins). Cone-beam computed tomography (CBCT) scans were carried out prior and after wound closure. The horizontal thickness of the augmentation at the buccal implant shoulder, procedure duration, and difficulty of each GBR procedure were recorded.

**Results:**

No statistically significant differences for the horizontal thickness of the augmentation at the buccal implant shoulder were obtained (*p*=0.8652). For the device, the median measured 2.35 mm (first quartile [Q1]: 2.03; third quartile [Q3]: 2.68) and and for L-shape it was 3.05 mm (Q1:2.48; Q3:3.23). The amount of time for the procedure was statistically significantly lower with 149.5 s using the device (Q1:127.25; Q3:160.5) compared to 206.5 s (Q1:198.75; Q3:236.75) using L-shape (*p*=0.0039). Difficulty was statistically significantly lower for the device with 2 (Q1:1; Q3:3) compared to 5 (Q1:4; Q3:5.25) for L-shape (*p*=0.0020).

**Conclusion:**

GBR with the device in combination with DBBM and a collagen membrane led to a similar amount of horizontal thickness of the augmentation as an L-shaped DBBM-C with a collagen membrane and fixation pins. The procedure with the device was significantly faster and easier for the clinician.

## 1. Introduction

When oral implantology was still in its infancy, the decision of the right position of a dental implant was driven by the amount of bone available in the particular region [1]. Based on esthetic considerations, this early concept changed to a prosthetically driven implant placement [2, 3]. A lack of bone due to congenital, postinflammatory, or postextraction ridge alterations often leads to fenestration and dehiscence-type-defects and therefore inadequate bone dimensions when performing a prosthetically oriented implant placement [4–8]. There is strong evidence documenting that the survival rates of dental implants placed simultaneously with guided bone regeneration (GBR) are in the same range as survival rates of dental implants placed in the native bone [9–13]. The technique of GBR requires the use of a membrane in combination with autologous bone or a bone substitution material, whereby the purpose of the membrane is to prevent ingrowth of rapidly proliferating soft tissue cells into the augmented area [1, 4, 14, 15]. A membrane either requires mechanical stability or does need support by the underlying bone substitute material to maintain space for new bone formation. There are two kinds of membranes used for GBR: resorbable and nonresorbable membranes. Titanium-reinforced polytetrafluorethylene (PTFE) membranes are the most commonly used nonresorbable membranes and provide sufficient stability, rigidity, and show good clinical long-term outcomes [7, 10, 16–20]. However, the major disadvantages of nonresorbable membranes are the need of a second surgical intervention to remove the membrane, and they have an increased risk for soft tissue dehiscences. Therefore, resorbable membranes have been developed and brought to the market [16, 21]. A systematic review and meta-analysis of 28 publications showed that the most often used type of intervention of lateral bone augmentation performed simultaneously with dental implant placement was a xenogeneic particulated grafting material and a resorbable collagen membrane and led to a mean defect resolution of 81.3% at re-entry [22]. Due to the fact that these mainly alloplastic and xenogeneic resorbable membranes lack the dimension stability and space maintenance of PTFE membranes, the support of a bone substitute material or underlying bone is required [22–26]. Even if these factors are taken into account, compression of the underlying material and displacement of the membrane itself can occur, as in vitro studies demonstrated, therefore highlighting the factor of flap closing and suturing [27–29]. In order to improve the volume stability of GBR procedures in combination with a resorbable membrane, the so-called “L-shape” technique was introduced [30]. This encompasses a soft-type substitute block, trimmed into an “L-shape” to be placed buccally and crestally at the implant to cover the defect. The membrane is further stabilized with pins on the buccal side and is finally pushed underneath the flap at the oral side. The supposed advantage of this technique is an increased stability of the augmented volume at the buccal and occlusal aspect of the dental implant. The technique might be limited to class I–III defects [15, 28, 30]. Especially the placement of pins remains clinically challenging. Therefore, this study aims to investigate an alternative that should ideally reach the same stabilization in combination with a facilitated application. The implant itself might be used to gain more stability at the buccal aspect. The advent of medical-grade bioresorbable materials such as copolymer polyglycolide-colactide (PGLA) and continuously improving manufacturing technologies might also open new possibilities for GBR procedures. The aim of the present study was therefore to develop, produce, and test (in vitro) a novel device to provide volume stability of the augmentation in GBR procedures at implant dehiscence bone defects and to evaluate a possible alternative to a current state of the art technique [27, 28, 30].

## 2. Materials and Methods

### 2.1. Novel Bioresorbable Device: Design, Material, and Production

**Figure 1:**
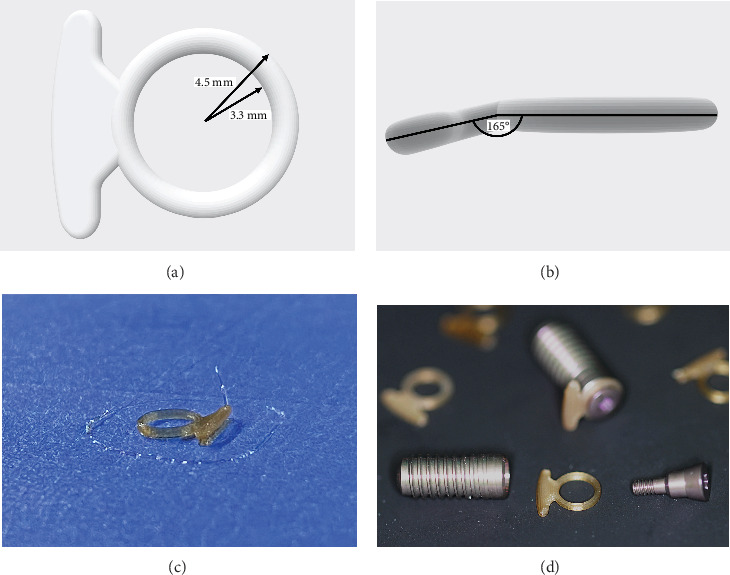
Design and production of the novel bioresorbable device. (a) The design consists of a ring with an inner- and outer diameter of 3.3 and 4.5 mm as well as an (b) 165° angled umbrella-like outrigger. (c) The 3D-printed PLGA medical-grade bioresorbable device. (d) The device will be mounted between the implant shoulder and the closure screw.

The device was designed using a computer-aided design (CAD) software (Fusion 360, Autodesk Inc., San Francisco, U.S.A.). It is composed of a ring and a directly attached umbrella-like two-armed outrigger. The inner and outer diameter of the ring amount 3.3 and 4.5 mm, respectively. The outrigger is attached in a 165° angle (Figure 1). The production was performed using a three-dimensional (3D) printer (Raise3D Pro2, Raise3D Inc., Rome, Italy) functioning with the additive manufacturing 3D printing technology of fused deposition modeling (FDP). The material of the device is 95/5 PGLA, a medical-grade bioresorbable copolymer based on established and Federal Drug Administration (FDA)-approved polymers. It further meets all the guidelines set forth by the Biological Test for Plastics, USP Class VI, and the acceptance criteria of ISO 10993-5 cytotoxicity testing. The degradation times of the material are, according to the manufacturer, subject to change based on device geometry and anatomical location and provides a strength retention of 0.25–4 weeks and showes mass loss in 1–3 months. For the 3D printing, a 1.75 mm diameter PGLA bioresorbable filament (MaxPrene 955, Poly-Med Inc., Anderson, SC, U.S.A.) was used in combination with a 0.2 mm extrusion nozzle. The starting layer height was 0.1 mm followed by a general layer height of 0.05 mm. The general extrusion width was 0.2 mm according to the nozzle diameter of 0.2 mm. The infill density was defined as 100% with 20 bottom and top solid fill layers each. The heat bed temperature was set at 60°C in combination with an extruder temperature of 215°C. The first layer printing speed was 15.0 mm/s followed by a general printing speed of 25.0 mm/s. Infill and solid fill speed were both 25.0 mm/s beside a travel *x*/*y* axis movement speed of 100.0 mm/s, a *z* axis movement speed of 5.0 mm/s, and a raft *x*/*y* axis movement speed of 100.0 mm/s.

### 2.2. In Vitro Model

**Figure 2:**
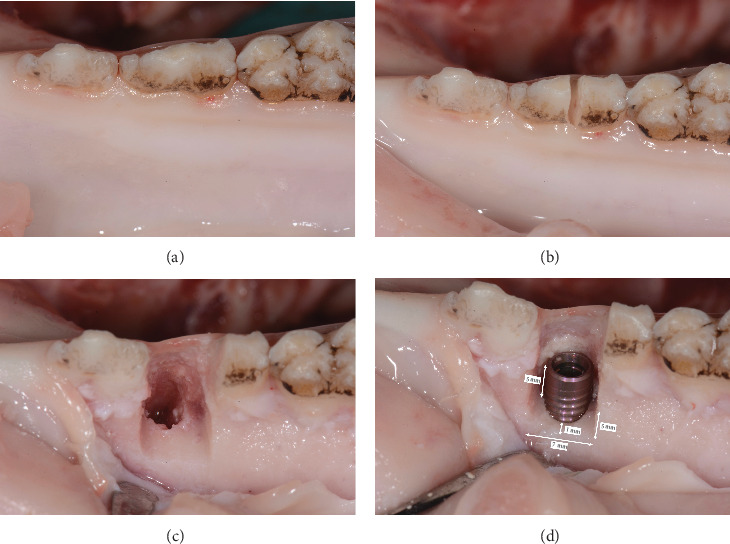
Preparation of the buccal bone dehiscence defect. (a) Initial anatomical situation of the lower pig jaw. (b) Hemisection of the second premolar and extraction of the distal root. (c) Elevation of mucoperiosteal flap and preparation of the box-shaped defect. (d) Inserted titanium implant.

Ten fresh pig mandibles were obtained from the local butcher from pigs aged 5 months. For this kind of study design, no approval of the competent ethics committee is needed. Second premolars were hemisectioned and their mesial roots were extracted. Crestal incisions were bilaterally performed mesial to the second premolars extraction sockets, and one vertical releasing incision was made at the distobuccal aspect of each second premolar. Mucoperiosteal flaps were elevated. Twenty box-shaped bone defects, one at each extraction site, were prepared by means of cylindrical carbide drills. The bone defects measured 7 mm mesiodistally, 5 mm buccoorally, and 5 mm apicocoronally (Figure 2). One 10 mm-length and 4.1 mm-diameter bone level titanium implant (Straumann bone level RC–Regular CrossFit, Straumann Holding AG, Basel, Switzerland) was inserted into each bone defect by placing the implant central axis along the lingual bone wall at the same distance from the mesial and the distal walls of the defect. The apicocoronal position of the implant shoulder corresponded to the most coronal part of the lingual bone wall. The distance between the implant surface and the most buccal aspect of the apical bone wall, in a direction perpendicular to the axis of the implant, measured 1 mm (Figure 2).

### 2.3. GBR and Wound Closure

**Figure 3:**
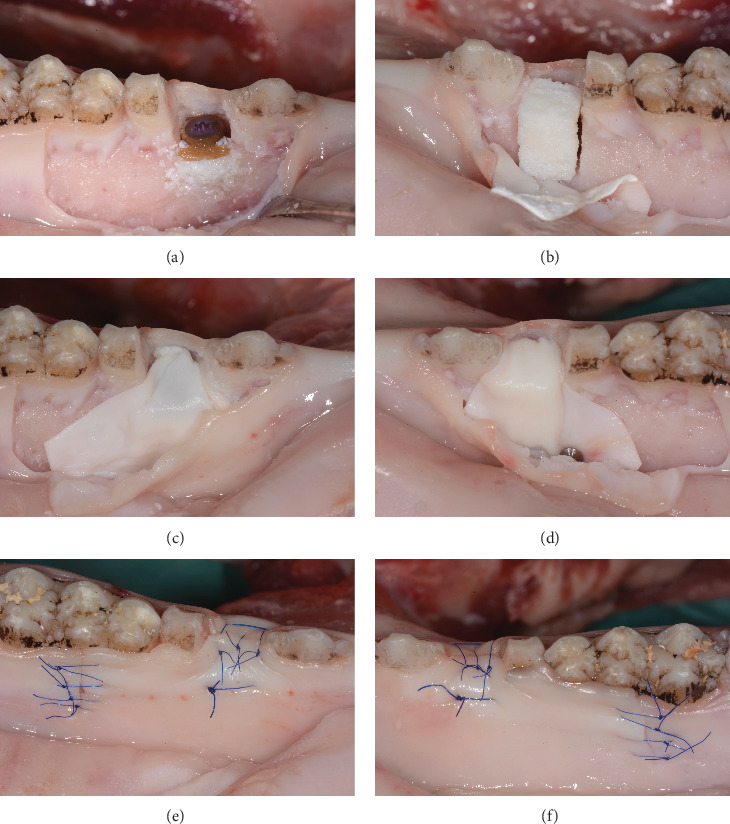
GBR procedures. (a) GBR procedure with the novel device and the (b) L-shape. (c) Collagen membrane without fixation pins covering the device and the (d) s-shape with use of fixation pins. (e) Flap closure of the GBR procedure with the device and with the (f) L-shape.

Each bone defect (*n* = 20) was augmented according to the randomization sheet. Hence, the following two GBR procedures (Figure 3) were randomly assigned by a randomization list made by an uninvolved person and allocated in opaque envelopes:

• Device (test): Particulated deproteinized bovine bone mineral (DBBM) (Geistlich Bio-Oss granules 0.25–1 mm, Geistlich Pharma AG, Wolhusen, Switzerland) + novel bioresorbable device (MaxPrene 955, Poly-Med Inc., Anderson, SC, U.S.A.) + collagen membrane (Geistlich Bio-Gide, Geistlich Pharma AG, Wolhusen, Switzerland) (*n* = 10).
• L-shape (control): L-shaped soft-block composed of 90% DBBM granules stabilized with 10% collagen (DBBM-C) (Geistlich Bio-Oss Collagen, 100 mg, 0.4–0.5 cc, Geistlich Pharma AG, Wolhusen, Switzerland) + collagen membrane (Geistlich Bio-Gide, Geistlich Pharma AG, Wolhusen, Switzerland) + two titanium fixation pins (Straumann Holding AG, Basel, Switzerland) (*n* = 10).

In the test group (device), the device was positioned on the implant and fixed with a 0.5 mm height closure screw (Straumann Holding AG, Basel, Switzerland). DBBM was used from buccally to cover the buccal surface of the implant and to recontour alveolar ridge. The area was then covered with a collagen membrane overlapping the walls of the defect by at least 2 mm (Figure 3).

In the control group (L-shape), a rectangular piece of DBBM-C was cut into an L-shaped block with a N°15 surgical blade. A periodontal probe was used to ensure a homogenous thickness of 3 mm in the occlusal portion and 2 mm in the buccal portion of the L. A collagen membrane was applied and stabilized by means of two titanium pins (Straumann Holding AG, Switzerland) placed 1 mm apically to the apical wall of the defect overlapping the walls of the defect by at least 2 mm (Figure 3).

A periosteal releasing incision was performed in the apical portion of the mucoperiosteal flap in both groups. The flaps were sutured with a polyamide monofilament suture (Dafilon 5–0, B. Braun Medical AG, Sempach, Switzerland). The surgeons performed a standard suturing procedure (one horizontal mattress and single interrupted sutures) (Figure 3).

### 2.4. Cone-Beam Computed Tomography (CBCT) Scanning and Image Evaluation

**Figure 4:**
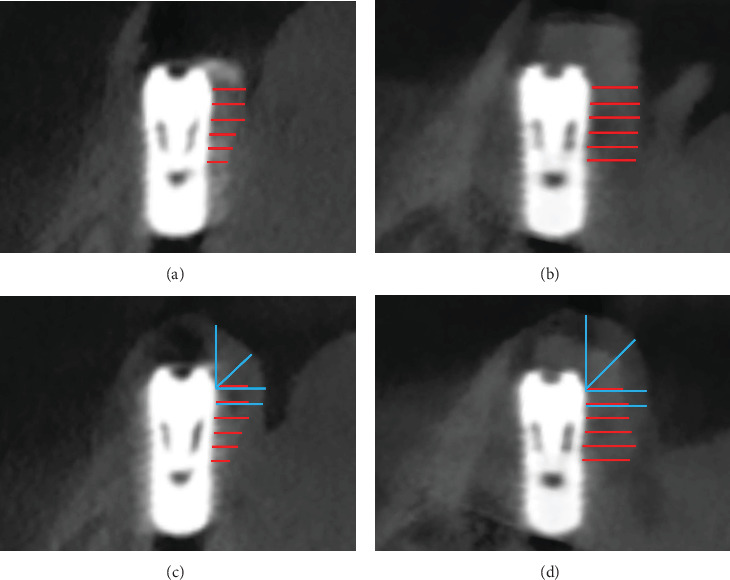
Bucco-oral CBCT reconstructions with the measurements of the augmented regions (pre-HT_0−5 mm_ and post-HT_0−5 mm_; red) and the overall volume from the buccal implant shoulder (post-H-STT_0−1 mm_, post-45-STT, post-V-STT; blue): (a) device and (b) L-shape GBR procedures before suturing, (c) device and (d) L-shape GBR procedures after suturing.

CBCT scans (Axeos, Dentsply Sirona, Charlotte, U.S.A.) of the pig mandibles were performed before and after the suturing of the mucosal flap (Figure 4). The lower jaws were placed on the supporting plate of the scanner. They were positioned with the occlusal plane parallel to the horizonal plane and in the center of the field of view (FOV) based on the red laser orientation beams. The CBCT scans were then obtained with the following technical parameters: 85 kV acceleration voltage, 10 mA beam current, FOV of 17 x 13 cm, 360° rotation, voxel size of 0.04 mm, and scan time of 5.9 s.

Every CBCT Dataset (Digital Imaging and Communications in Medicine [DICOM]) was evaluated with an imaging software (Synedra View, Synedra, Innsbruck, Austria). A digital template representing all relevant dimensional implant parameters was used for reproducibility of the measurements. Hard tissue parameters included the horizontal thickness levels of the augmentation at 0–5 mm distance from the implant shoulder before and after suturing (pre-HT_0–5 mm_ and post-HT_0–5 mm_). Soft tissue parameters included the overall horizontal soft and hard tissue thickness at 0–1 mm distance from the implant shoulder after suturing (post-H-STT_0–1 mm_), the overall 45° soft and hard tissue thickness from the implant shoulder (post-45-STT) and the overall vertical soft and hard tissue thickness from the implant shoulder (post-V-STT) (Figure 4).

### 2.5. Length of the Procedure and Difficulty of the Procedure

For every GBR procedure, the overall duration in seconds to perform the GBR procedure was assessed (GBR-time). After every GBR procedure, the difficulty of each procedure was rated from 0 (very easy) to 10 (very difficult) by a numerical rating scale (NRS). Two experienced surgeons (Stefan Bienz P and Riccardo Kraus D) performed the experimental surgical interventions, each one treating the same amount of sites. Calibration was performed by conducting the surgery at one additional cadaver for each surgeon, which is not included in the analysis.

### 2.6. Sample Size Calculation and Statistical Analysis

Sample size calculation was performed for two independent groups with a continuous primary endpoint. Alpha was set at 5%, beta at 20%, and power at 80%. Sample size was four for the test group and four for the control group with a total of eight. Data were recorded in a spreadsheet (Microsoft Excel, Microsoft Corporation, Redmond, Washington, USA) and statistical analysis was performed with a statistical analysis software (SAS 9.4, SAS Corp., Cary, NC, USA). Descriptive summary statistics for numerical variables was obtained and intergroup comparison between the medians of the test and control group was performed by Wilcoxon-signed-rank test. The level of significance was set at 5%. Corrections for the multiple testing were not applied since only one primary endpoint post-HT_0 mm_ was considered.

## 3. Results

All implants were placed in the planned position inside the created defect and sufficient stability was achieved in order to perform the subsequent manipulations. All GBR procedures in the test and control group were performed successfully according to the protocol. Tension-free primary closure of the mucoperiosteal flap was also achieved in all sites.

### 3.1. Hard Tissue Analysis

For the primary outcome, horizontal thickness of the augmentation at the buccal implant shoulder after suturing (post-HT_0 mm_), the median measured 2.35 mm (first quartile [Q1]: 2.03; third quartile [Q3]: 2.68) for the device and 3.05 mm (Q1:2.48; Q3:3.23) for L-shape and was not statistically significantly different (*p*=0.8652). Of all horizontally measured values between HT_0 mm_ and HT_5 mm_, the lowest were 1.8 mm (Q1:1.30; Q3:2.60) for pre-HT_5 mm_ (device) and 1.8 mm (Q1:1.60; Q3:2.80) for post-HT_5 mm_ (device) and the highest was 3.3 mm (Q1:2.58; Q3 : 4.33) for pre-HT_5 mm_ (L-shape). None of the differences was statistically significant. All values before and after suturing between HT_0 mm_ and HT_5 mm_ are summarized in Table 1. In terms of the vertical height of the buccal grafting material, all sites in both groups covered the buccal implant shoulder completely before and after suturing.

### 3.2. Overall Volume From the Buccal Implant Shoulder

The overall horizontal soft and hard tissue thickness after suturing (post-H-STT_0 mm_) amounted to 3.85 mm (Q1:3.28; Q3:4.75) for the device, and 4.30 mm (Q1:3.75; Q3:5.00) for L-shape (*p*=0.1621). The overall thickness measured in a 45° angle from the buccal implant shoulder (post-45-STT) was lower for the device (3.35 mm [Q1:2.88; Q3:3.80]) as compared to L-shape (4.10 mm [Q1:3.68; Q3 : 4.65]) (*p*=0.0156). The overall vertical thickness measured from the buccal implant shoulder (post-V-STT) was lower for the device (3.40 mm [Q1:2.98; Q3:4.43]) as compared to L-shape (5.15 mm [Q1:4.48; Q3:5.48]) (*p*=0.0273). All values are summarized in Table 2.

### 3.3. Duration of the Procedure and Difficulty of the Procedure

The overall time of the GBR procedure (GBR-time) was 149.5 s (Q1:127.25; Q3:160.5) for the device and 206.5 s (Q1:198.75; Q3:236.75) for L-shape and was statistically significantly lower for the device (*p*=0.0039). The median NRS amounted to 2 (Q1:1; Q3:3) for the device and 5 (Q1:4; Q3:5.25) for L-shape and was statistically significantly lower for the device (*p*=0.0020).

## 4. Discussion

The present study revealed: (i) A similar amount of horizontal thickness of the augmentation and a complete vertical fill for both groups. (ii) A higher volume gain with L-shape above the buccal implant shoulder. (iii) Performance of a GBR procedure with the device was faster. (iv) Performance of a GBR procedure with the device was easier for the clinician.

The present study comprised the development, production, in vitro testing, and CBCT data analysis of a novel bioresorbable device. The goal of this device was to provide volume stability of the augmentation in GBR procedures at implant dehiscence bone defects and to evaluate a possible alternative to a current state of the art GBR technique [27, 28, 30]. It was successfully demonstrated that the horizontal thickness of the augmentation at the implant shoulder after suturing (post-HT_0 mm_) was not statistically significantly different from the device and the L-shape technique. It was shown that a complete vertical fill of the buccal grafting material up to the implant shoulder could be achieved for both groups before and after suturing. As shown in a previous in vitro study, manipulation of mucosal flaps during suturing after GBR can lead to a displacement of the bone substitute and a partial collapse of the collagen membrane [27]. The same study further demonstrated that the stability of the bone substitute and collagen membrane was enhanced by the use of fixation pins [27]. Another study of the same research group showed that GBR with an L-shaped DBBM-C in combination with a particulate xenogeneic bone substitute, a collagen membrane and fixation pins significantly improved the horizontal volume stability of implant dehiscence bone defects compared to a particulate bone substitute with a collagen membrane and fixation pins [28]. On the one hand, these previous in vitro studies indicate that the addition of fixation pins appears to play an advantageous role in terms of the stability of GBR procedures. On the other hand, the clinical application of fixation pins represents a demanding and potentially time-consuming surgical step [31, 32]. At this point, it is important to highlight that in the present study, no fixation pins were used for GBR procedures with the device. The results showed that for GBR procedures with the novel device, no fixation pins are needed to achieve similar amount of horizontal thickness of the augmentation.

The present study further showed a statistically significantly higher volume gain above the buccal implant shoulder for GBR with the L-shape technique than with the device. Apparently, the higher volume gain of the L-shape GBR procedure led to the requirement of a higher soft tissue mobilization in order to achieve a tension-free flap-closure [30]. In this context, it should be kept in mind that augmented bone above implant shoulder level will be resorbed before or shortly after prosthetic rehabilitation [4, 33, 34]. A bone augmentation above the device was obviously not possible. Hence the overall 45° thickness and the overall horizonal thickness were statistically significantly lower when performing GBR procedures with the device.

The required time for the performance of a GBR procedure with the device was statistically significantly shorter than for the L-shape. The shorter duration of the GBR procedure with the device attributed to the fewer and shorter working steps involved in the entire GBR treatment. The placement of the bone substitute and application of the membrane without fixation pins was faster.

To assess the difficulty of the different GBR procedures, every procedure was rated from 0 (very easy) to 10 (very difficult) on a NRS. The GBR procedure with the device was statistically significantly easier than the L-shape procedure. The device was fixed with the closure screw on the implant shoulder. It rigidly held its distinct position on the implant shoulder and stabilized the augmentation with its umbrella-like outrigger. This step was followed by the application of the bone substitute material on the implant bone dehiscence defect and the coverage with a collagen membrane. The working steps of cutting a piece of DBBM-C into an L-shape and placement of fixation pins were obsolete in the GBR procedure with the device.

Current possibilities of manufacturing with highly innovative CAD/CAM technologies in combination with medical-grade bioresorbable polymers are likely to offer new possibilities, especially in the field of GBR procedures [35–39]. The idea of a device consisting of a ring with an attached outrigger being able to be mounted between the implant shoulder and the closure screw in order to stabilize the augmented volume at implant dehiscence bone defects is considered as an interesting approach for GBR procedures.

As the present study was performed in vitro, several questions remain unanswered. It only outlined the application and feasibility, without any information in regard to healing, degradation, and final volume gain. The fact that a medical-grade bioresorbable polymer was used to produce the device underlines primarily the intention and efforts of exploring a new concept for clinical GBR procedures that could be further tested in in vivo situations. For the purpose of performing GBR clinically, the bioresorption time and mechanical integrity of a bioresorbable device should ideally match the biological periods of tissue formation [40]. Therefore, the mechanical integrity of an ideal device should optimally be maintained up to 6 weeks and resorption should be completed at 3 months. Many clinical concepts suggest to continue with soft tissue grafting or abutment connection after 3 months. Remnants of the device at this time-point could cause infections once there is a communication with the oral cavity. Current polymers with resorption rates in this range exist. However, another big challenge is currently seen in inflammatory effects of bioresorbable polymers on soft tissue healing and bone formation. The release of oligomers and acid byproducts during degradation cause inflammatory foreign-body reactions and might negatively affect the outcome. This was demonstrated in studies investigating PLGA-based membranes, although the membranes were noncytotoxic and biodegradable [15, 41, 42]. Focusing on intraoral biological requirements, despite the in vitro setting of the present study, an already on the market available medical-grade bioresorbable PGLA copolymer (MaxPrene 955, Poly-Med Inc., Anderson, SC, U.S.A.) was selected to produce the device. According to the manufacturer, this PGLA bioresorbable polymer provides full strength retention of 0.25–4 weeks and shows mass loss in 1–3 month, depending on processing, physiological environment, and anatomical location in the human body. In vivo studies showed that degradation of synthetic polymers begins with large molecular chains followed by short chains in the backbone and the hydrolysis of amorphous regions, rather than enzymatic degradation [43]. A comparable method to provide in vivo conditions, uses alkaline medium, including sodium hydroxide (NaOH) solutions, which is rich in hydroxyl (-OH) groups, to increase hydrolysis [44]. In vitro studies investigating the degradation behavior of bioresorbable polymers often use the method of accelerated degradation [45]. Due to the lack of evidence regarding bioresorptive characteristics of PGLA under intraoral conditions, further specific in vivo testing simulating an oral environment are required.

Despite the mentioned limitations, this setup nonetheless allowed for the standardization of the surgical steps of each GBR procedure such as bone defect preparation, amount of applied bone substitute material, as well as design and suturing of the mucoperiosteal flaps. The present study emphasizes the development of a clinically testable bioresorbable device for further in vivo testing.

## 5. Conclusions

In an in vitro setup, GBR with the device in combination with DBBM and a collagen membrane led to a similar amount of horizontal thickness of the augmentation as an L-shaped piece of DBBM-C with a collagen membrane and fixation pins. The procedure with the device was significantly faster and easier for the clinician.

## Figures and Tables

**Table 1:** Hard tissue parameters.

Group	Variable	*N*	Mean	SD	Min	Q1	Median	Q3	Max
Device	Pre-HT_0 mm_	10	2.45	0.6	1.7	2.03	2.35	2.68	3.7
L-shape	Pre-HT_0 mm_	10	2.92	0.46	2.1	2.48	3.05	3.23	3.6
Device	Post-HT_0 mm_	10	2.32	0.56	1.7	1.8	2.2	2.65	3.4
L-shape	Post-HT_0 mm_	10	2.34	0.68	1.2	1.9	2.35	2.75	3.6
Device	Pre- HT_1 mm_	10	2.59	0.59	1.8	2.08	2.5	3.03	3.6
L-shape	Pre-HT_1 mm_	10	3.06	0.58	2	2.55	3.15	3.48	3.8
Device	Post-HT_1 mm_	10	2.51	0.57	1.8	2.05	2.35	3.03	3.4
L-shape	Post-HT_1 mm_	10	2.5	0.58	1.5	2.08	2.55	2.9	3.4
Device	Pre-HT_2 mm_	10	2.64	0.68	1.4	2.15	2.55	3.28	3.5
L-shape	Pre-HT_2 mm_	10	3.12	0.62	2	2.75	3.1	3.5	4.2
Device	Post-HT_2 mm_	10	2.54	0.64	1.6	2	2.4	3.08	3.6
L-shape	Post-HT_2 mm_	10	2.77	0.7	1.6	2.28	2.85	3.15	4
Device	Pre-HT_3 mm_	10	2.55	0.72	1.1	2.03	2.65	3.23	3.4
L-shape	Pre-HT_3 mm_	10	3.08	0.57	2.4	2.68	3	3.38	4.2
Device	Post-HT_3 mm_	10	2.58	0.78	1.5	2	2.4	3.03	4.2
L-shape	Post-HT_3 mm_	10	3.08	0.88	1.7	2.28	3.1	3.55	4.7
Device	Pre-HT_4 mm_	9	2.29	0.79	1	1.55	2.3	2.95	3.4
L-shape	Pre-HT_4 mm_	8	3.14	0.78	1.6	2.9	3.2	3.65	4.3
Device	Post-HT_4 mm_	9	2.2	0.95	0.8	1.3	2.1	3.15	3.4
L-shape	Post-HT_4 mm_	10	3.21	0.83	1.5	2.75	3.2	3.8	4.4
Device	Pre-HT_5 mm_	7	2.09	0.96	1	1.3	1.8	2.6	3.8
L-shape	Pre-HT_5 mm_	4	3.4	0.92	2.4	2.58	3.3	4.33	4.6
Device	Post-HT_5 mm_	7	2.13	0.9	1.1	1.6	1.8	2.8	3.8
L-shape	Post-HT_5 mm_	7	2.99	1.13	1.2	1.7	3.3	3.9	4

*Note*: Descriptive data for all evaluated hard tissue variables. Q1 = 25% quartile, Q3 = 75% quartile, pre-HT_0−5 mm_ = horizontal thickness levels of the augmentation at 0–5 mm distance from the implant shoulder before suturing, post-HT_0−5 mm_ = horizontal thickness levels of the augmentation at 0–5 mm distance from the implant shoulder after suturing.

Abbreviations: Max, maximum; Min, minimum; *N*, number; SD, standard deviation.

**Table 2:** Soft tissue parameters.

Group	Variable	*N*	Mean	SD	Min	Q1	Median	Q3	Max
Device	Post-H-STT_0 mm_	10	3.94	0.81	2.7	3.28	3.85	4.75	5
L-shape	Post-H-STT_0 mm_	10	4.43	0.93	3.2	3.75	4.3	5	6.1
Device	Post-H-STT_1 mm_	10	4.32	0.87	3.4	3.48	4.2	5.25	5.6
L-shape	Post-H-STT_1 mm_	10	4.72	0.92	3.7	3.98	4.55	5.08	6.8
Device	Post-45-STT	10	3.33	0.56	2.6	2.88	3.35	3.8	4.4
L-shape	Post-45-STT	10	4.28	0.97	2.9	3.68	4.1	4.65	6.5
Device	Post-V-STT	10	3.79	1.02	2.7	2.98	3.4	4.43	6
L-shape	Post-V-STT	10	4.95	0.84	3	4.48	5.15	5.48	6

*Note:* Descriptive data for all evaluated soft tissue variables. Q1 = 25% quartile, Q3 = 75% quartile, post-H-STT_0−1 mm_ = overall horizontal soft and hard tissue thickness at 0–1 mm distance from the implant shoulder after suturing, post-45-STT = overall 45° soft and hard tissue thickness from the implant shoulder, and post-V-STT = overall vertical soft and hard tissue thickness from the implant shoulder.

Abbreviations: Max, maximum; Min, minimum; *N*, number; SD, standard deviation.

## Data Availability

The data that support the findings of this study are available from the corresponding author upon reasonable request.
